# Generation of Aptamers from A Primer-Free Randomized ssDNA Library Using Magnetic-Assisted Rapid Aptamer Selection

**DOI:** 10.1038/srep45478

**Published:** 2017-04-03

**Authors:** Shih-Ming Tsao, Ji-Ching Lai, Horng-Er Horng, Tu-Chen Liu, Chin-Yih Hong

**Affiliations:** 1 Institute of Biochemistry, Microbiology and Immunology, Chung Shan Medical University, Taichung, Taiwan; 2 Sections of Infectious Diseases, Department of Internal Medicine, Chung Shan Medical University Hospital, Taichung, Taiwan; 3 Institute of Electro-optical Science and Technology, National Taiwan Normal University, Taipei, Taiwan; 4 Research Assistant Center, Chang Hua Show Chwan Health Care System, Changhua, Taiwan; 5 Department of Chest Medicine, Cheng-Ching General Hospital, Taichung, Taiwan; 6 Graduate Institute of Biomedical Engineering, National Chung Hsing University, Taichung, Taiwan

## Abstract

Aptamers are oligonucleotides that can bind to specific target molecules. Most aptamers are generated using random libraries in the standard systematic evolution of ligands by exponential enrichment (SELEX). Each random library contains oligonucleotides with a randomized central region and two fixed primer regions at both ends. The fixed primer regions are necessary for amplifying target-bound sequences by PCR. However, these extra-sequences may cause non-specific bindings, which potentially interfere with good binding for random sequences. The Magnetic-Assisted Rapid Aptamer Selection (MARAS) is a newly developed protocol for generating single-strand DNA aptamers. No repeat selection cycle is required in the protocol. This study proposes and demonstrates a method to isolate aptamers for C-reactive proteins (CRP) from a randomized ssDNA library containing no fixed sequences at 5′ and 3′ termini using the MARAS platform. Furthermore, the isolated primer-free aptamer was sequenced and binding affinity for CRP was analyzed. The specificity of the obtained aptamer was validated using blind serum samples. The result was consistent with monoclonal antibody-based nephelometry analysis, which indicated that a primer-free aptamer has high specificity toward targets. MARAS is a feasible platform for efficiently generating primer-free aptamers for clinical diagnoses.

Systematic Evolution of Ligands by EXponential enrichment (SELEX) was originally developed by three separate groups during the early 1990s^1^,^2^,^3^. The SELEX protocol is an *in vitro* selection method for isolating DNA or RNA sequences that bind to a specific target. These DNA or RNA sequences or aptamers can be produced through chemical synthesis. Aptamers can also be modified for biological applications^4^,^5^,^6^,^7^, for application in situations traditionally addressed by antibodies^8^. Currently, there are potential aptamers undergoing clinical trials^9^,^10^,^11^,^12^. For example, a treatment for age-related macular degeneration, Macugen (EyeTech Pharmaceuticals, New York, NY, USA), has been approved by the United States Food and Drug Administration.

The basic steps of the SELEX process are incubation, separation, elution, amplification, and single-strand oligonucleotide purification; this is usually repeated for several rounds. Usually, between five and fifteen SELEX selection rounds must be performed until no further enrichment of functional nucleic acid species is detectable and the dissociation constants (K_d_) of low-micromolar to low-nanomolar are achieved. Most aptamers identified through a standard or modified SELEX procedure begin with a substantial random library pool. The random library contains random nucleotides at the central region and two fixed regions at the 5′ and 3′ ends of the oligonucleotide. The length of random sequences is approximately 20–60 bases and the fixed region is 20 nt at the 5′ and 3′ ends. Accordingly, for the SELEX process, the total length of oligonucleotides in the library is approximately 60–100 nt. The advantage of using fixed regions is that it serves as primers of target-bound sequence during PCR amplification. However, primers may cause non-specific binding, which may result in a false binding sequence or interfere with binding within random sequences^13^,^14^,^15^.

There were several studies conducted to restrict binding biases caused by these fixed regions, which includes: primer-annealing and primer-switching genomics SELEX^16^, primer free 2′-O-methyl random RNA fishing SELEX^17^, primer-less random RNA library selection: tailored-SELEX^18^, dual random RNA library selection: dual SELEX protocol^19^, minimal-primer random DNA library selection^20^,^21^, modified nucleoside triphosphates for *in vitro* selection^22^. However, all of the primer-free or minimal-primer approaches included several complicated molecular cloning steps for every selection cycle, which were time and resource intensive. These additional steps also introduced other biases that may interfere with results.

Recently, the Magnetic-Assisted Rapid Aptamer Selection (MARAS) method has been reported to generate high-binding affinity aptamers, in which the affinity of selected aptamers depends on the frequency and amplitude of externally applied magnetic fields^23^,^24^. The equilibrium dissociation constant of aptamer isolated using the MARAS reached a single digital nanomolar concentration through applying a proper frequency/strength of a rotating/alternating magnetic field. The MARAS method does not require the repeated selection cycles to produce high-affinity aptamers. As such, the MARAS method is effective for developing primer-free aptamer selection protocol. In this study, a new primer-free method using a non-fixed region (primer free) library through a Rotating Magnetic Field MARAS (RO-MARAS) method was proposed and demonstrated. The RO-MARAS method directly generated real primer-free aptamers, which have high-binding affinity to targets. Moreover, the highly-specific binding of selected aptamers to targets was validated using blind clinical serums. Results were consistent with those of clinical assays using monoclonal antibody-based nephelometry analysis.

## Results and Discussion

### Characteristics of isolated aptamer

In this study, a new and efficient MARAS protocol was used to generate no-fixed primer region aptamers (PF-aptamers). The PF-aptamer isolated using the MARAS platform had high binding affinity and specificity and could be used for clinical diagnoses. Several PF-aptamers were isolated through RO-MARAS. The sequence of the PF-aptamer (PF20N-RO-MARAS-84-1) is listed in Table 1, and the sequences of the other aptamers are listed in Supplementary Information Table S1. The low yield of a suitable aptamer is attributed to the low success rate of single-strand ligation. Only one aptamer (PF20N-RO-MARAS-84-1) was selected and used for the following experiment. The secondary structure and free energy of the aptamer were analyzed using an Mfold algorithm web site^25^ (Supplementary Information Fig. S1). The secondary structures of the PF-aptamer tended to form a small loop structure. The free energies ΔG of PF-aptamer is −2.93 kcal/mol.

### Reverse validation of isolated PF-aptamer

**Figure 1:**
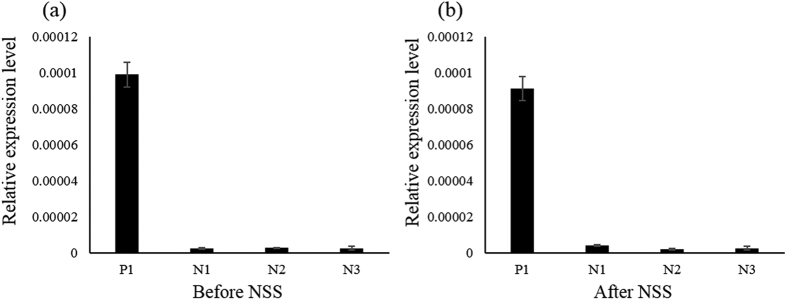
Results of the reverse validation of selected PF-aptamers (PF20N-RO-MARAS-84-1) with positive (CRP) and negative controls (N1, N2, and N3). (**a**) Before NSS, (**b**) After NSS, to provide nonspecific suppression.

The binding specificity of the selected aptamer was analyzed by reverse-targeting the positive control (P1) and negative controls (N1, N2 and N3). The result is shown in Fig. 1. The PF20N-RO-MARAS-84-1 aptamer bound to positive control MNPs and also to negative control MNPs before the application of nonspecific suppression (Before NSS) using the specific magnetic field condition as that used in aptamer selection stage (Fig. 1(a)). The level of binding toward the positive control was much higher than that of the negative control. After being subjected to nonspecific suppression (After NSS), the aptamer still bound to the positive control and negative controls, however at lower levels (Fig. 1(b)). The differences of the bound levels prior to and after nonspecific suppression can be attributed to the nonspecific suppression resulted from the stretch force acting on the bond between the aptamer and biomolecules other than the target. The stretch force is resulted from the motion of magnetic bound mixture in an aqueous solution induced by the interaction between the magnetic field and the magnetic dipole of the magnetic particle^23^,^24^. For negative controls, the levels of binding were low near the noise level. This result demonstrates that MARAS provides a competitive mechanism to dissociate non-specific and low-affinity bindings. Only high-specific and high-affinity bindings between selected aptamer-target bindings could not be interrupted after applying the MARAS mechanism.

### Dissociation constants of isolated PF-aptamers

The sequences of aptamer-dependent primers, as well as the target aptamer are listed in Table 1. In the q-PCR process, PF20N-RO-MARAS-84-1 was used as a template, combined with PF20N-RO-MARAS-84-1 Ex primer and PF20N-RO-MARAS-84-1 RR primer. Ex primer is an aptamer-specific stem-loop primer, 5′-GTCGTATCCAGTGCAGGGTCCGAGGTATTCGCACTGGATACGACcXn-3′ (Ex primer), that can form a stem-loop with a single-strand extension of additional n monomers (cXn) at the 3′ end. The monomers forming the stem loop are the same as the stem-loop primer used for single-strand ligation. The predicted secondary structure of Ex primer was shown in Supplementary Information Fig. S2. The reverse transcription and analysis of PF20N-RO-MARAS-84-1 was described in the material section. The apparent dissociation constant (K_d_) of the selected PF-aptamer was determined by q-PCR and fitted to the results with a non-linear regression. The fitting curve is shown in Supplementary Information Fig. S3 and the K_d_ of selected PF-aptamer is 23.58 ± 0.82 nM. For the purpose of comparing the affinity of aptamers with or without primers to the target^23^, it was found that the value of the dissociation constant of a primer-free aptamer is slightly higher than those with primers through RO-MARAS. This may be attributed to the reduction of the competitive mechanism provided by the MARAS method resulted from the decrease of aptamer size; 20 nt for PF-aptamer as opposed to 60 nt for aptamer with primers (20 nt for randomized sequence with 40 nt for two primers). Therefore, the quality (binding affinity to the target in terms of the dissociation constant) of an isolated aptamer using MARAS is only slightly deteriorated by using a PF library.

### Using PF-aptamer as a bio-probe in detecting bio-target

**Figure 2:**
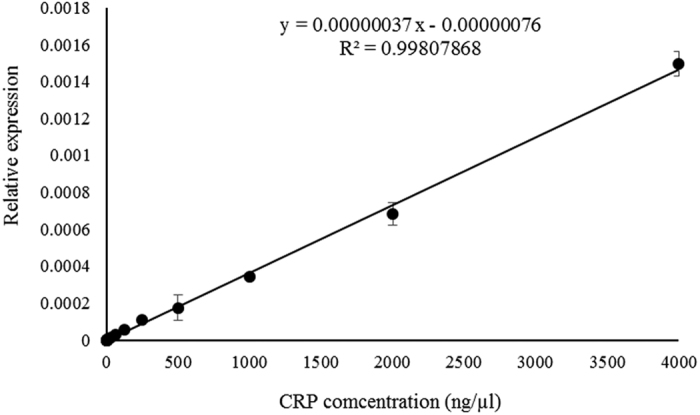
Standard calibration curve of CRP concentration using PF-aptamer as the capture probe.

**Figure 3:**
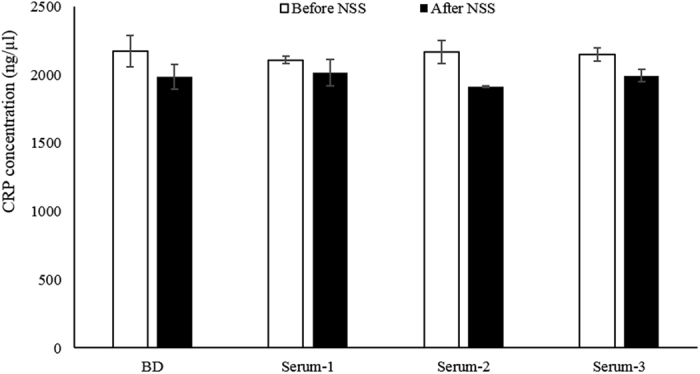
Recovery rate of spiked pure CRP of concentration 2000 nM in binding buffer, serum-1, -2, and -3 using PF-aptamer as the capture probe.

**Figure 4:**
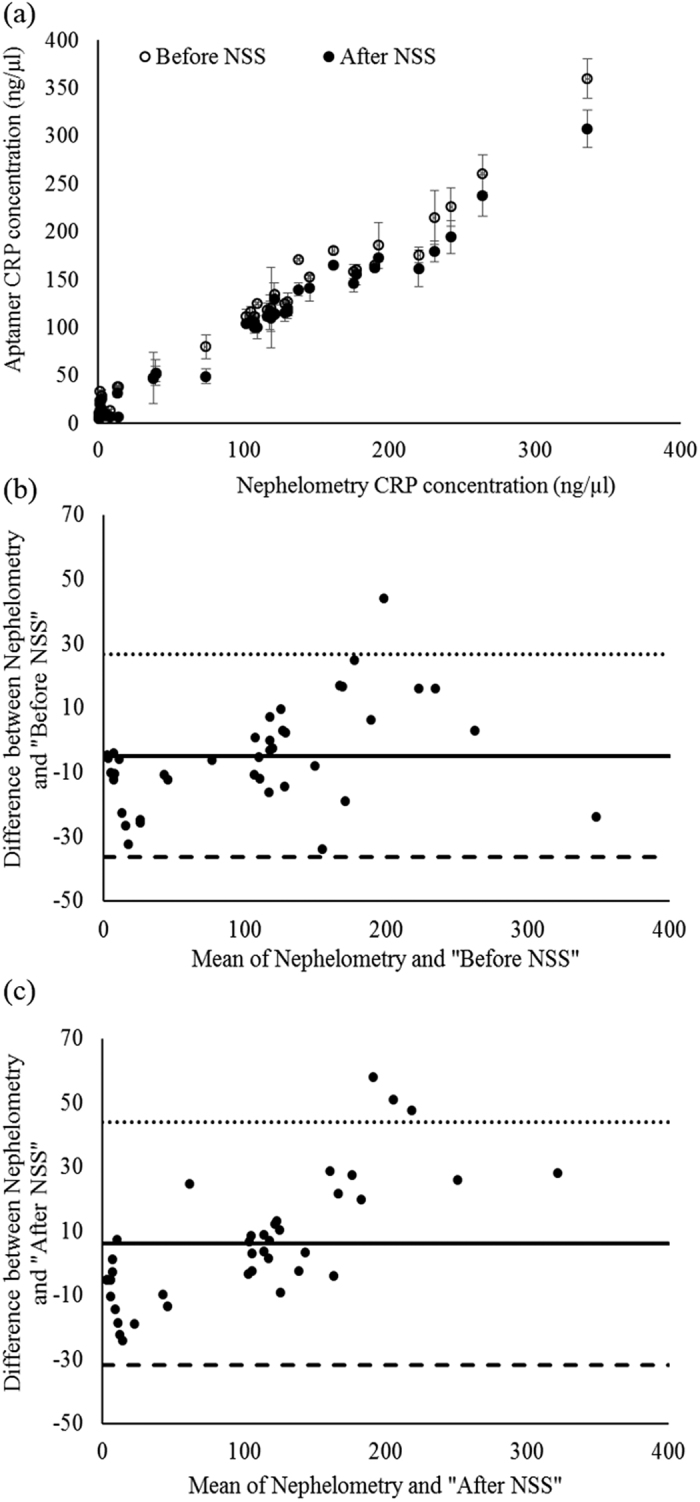
Comparison of the measured CRP concentration using PF-aptamer-based q-PCR method and monoclonal antibody based nephelometry method for blind serum samples. (**a**) The correction of CRP concentration between the two measurement methods; (**b**) and (**c**) Bland-Altman plot analyses of the CRP concentrations of nephelometry and “Before NSS” and “After NSS”, respectively.

The equation of the standard curve is linearly fitted from 16 q-PCR analyses, as shown in Fig. 2 with a linear equation and coefficient of determination. As expected, the relative expression level of q-PCR is linearly proportional to CRP concentration. Next, for analyzing the recovery rate of CRP using the PF-aptamer as a capture probe, mixed MNPs (spiked CRP in binding buffer, serum-1, -2, and -3 MNPs) were incubated with PF-aptamer; the detailed experiment is described in the method section. The amount of bound aptamer was analyzed by q-PCR and the concentrations of CRP were converted from the relative expression level of q-PCR through the standard calibration curve. The result is shown in Fig. 3. There is no significant difference among the CRP recovery rate in binding buffer, serum-1, -2, and -3. The hollow bars representing the CRP recovery rate for “Before NSS” were higher than those for “After NSS” (filled bar). Data indicated that the aptamer-CRP association was not interfered with by other proteins presented in serum samples. The lowest recovery rate of the spiked CRP protein is 95.5%. Finally, for the specificity analysis using the PF-aptamer as a detecting probe, the PF-aptamer was incubated with serum MNPs of forty blind serums and the amount of bound aptamer was analyzed by q-PCR. The results of CRP concentration in blind serums were compared with those using monoclonal antibody based nephelometry methods. As shown in Fig. 4(a), the hollow circles represent “Before NSS” CRP concentration in blind serum samples using PF-aptamer as the detecting probe and the filled circles represent the corresponding “After NSS” concentrations. Figure 4(a) illustrates the relationship between the CRP concentrations measured using PF-aptamer based q-PCR analysis (vertical axis) and the monoclonal antibody based nephelometry methods (horizontal axis). The results of “Before NSS” were higher than those of “After NSS” as expected, similar to the results of Fig. 3. A Bland-Altman plot^26^ was constructed to evaluate the level of analysis between the monoclonal antibody-based nephelometry method and the PF-aptamer based q-PCR method, including “Before NSS” and “After NSS” (Fig. 4(b) and (c)). Bland-Altman analysis revealed a bias of −4.89 (ng/μl) with 95% limits of measurement within the range 16.10–36.46 (ng/μl) for “Before NSS” and a bias of 6.18 (ng/μl) with 95% limits of measurement within the range 19.33–31.72 (ng/μl) for “After NSS”. Results indicate that the two analysis methods, PF-aptamer based q-PCR and monoclonal antibody based nephelometry, were highly consistent by analyzing CRP concentration in blind serum samples. The correlation analysis between PF-aptamer based q-PCR and monoclonal antibody based nephelometry analyses in all blind samples was performed (Spearman’s Rho = 0.985, *P* < 0.001 for “Before NSS”; Spearman’s Rho = 0.987, *P* < 0.001 for “After NSS”). This demonstrates that the application of the suppressing mechanism (a rotating magnetic field of 27 KHz and 14 gauss) has only a secondary effect on the measurement (Rho = 0.985 vs. Rho = 0.987). This may indicate that the application of suppressing mechanisms and the usage of magnetic particles during diagnosis may not be necessary. Therefore, the generated PF-aptamer can be used as a bio-probe in a variety of analysis methods commonly used in clinical immunoassays.

## Conclusion

Aptamers are known for high binding affinity, however a major obstacle of aptamer application for immunoassays is that the specificity might cause false positive results. In this study, a method based on MARAS technology was proposed and demonstrated to generate aptamers with high-binding affinity and high-specificity using a primer-free oligonucleotide library. The generated PF-aptamer has high-binding affinity and high-specificity toward a target molecule, which is validated by K_d_ and the recovery rate determined by q-PCR analysis using three blind samples, respectively. According to the experiments using forty blind serum samples, both results of PF-aptamer based q-PCR analyses with and without application of suppressive mechanisms using the MARAS method are consistent with those using monoclonal antibody-based nephelometry analysis. The strengths and limitations of primer-free RO-MARAS and other modified primer-free SELEX are listed in Table 2. It can be concluded that the MARAS method is effective for PF-aptamer generation in which the generated PF-aptamer can be used as a bio-probe to detect target molecules in clinical applications, even without the use of magnetic particles.

## Methods

### Oligonucleotide library and primer sequence

The DNA oligonucleotide library used in this study was chemically synthesized on a 1 mM scale and PAGE-purified (purchased from MDBio, Taipei, Taiwan). A 20-nt randomized oligonucleotide was used as a starting library (5′-N_20_-3′: PF library). A 5′-phosphorylation single-strand stem-loop primer (ds-5′-p-stem-loop-3′ primer): 5′-p-GGGGGGTCGTATCCAGTGCAGGGTCCGAGGTATTCGCACTGGATACGAC-3′ was used for single-strand ligation of isolated aptamers. The 5 G nucleotide monomers at the 5′ end extended the stem loop with a single-strand for single-strand ligation. The remaining sequence forms a stem loop to ensure that the sequence did not interfere with the single-strand ligation. Other types and lengths of stem-loop primer may also be used. A set of primers: 5′-AGTGCAGGGTCCGAGGT-3′ (PFFR), which is a partial sequence (underlined) of the stem-loop primer and 5′-CGACTCTAGAGGGGATCCAG-3′ (PFRR), which complemented a partial sequence in the yT&A-vector near the DNA insertion region, was used for PCR amplification. Another set of primers, 5′-GTTTTCCCAGTCACGAC-3′ (M13FR) and 5′-TCACACAGGAAACAGCTATGAC-3′ (M13 RR), was used to verify the cloning step. The universal T7 primer was used to sequence the nucleotide of the selected aptamer (T7: 5′-TAATACGACTCACTATAGGG-3′). These primers were purchased from MDBio.

### CRP and serum-protein coated bio-functionalized magnetic particles

In this study, human C-reactive proteins (CRP) with purities of greater than 99% were used as targets for positive selection (purchased from MYBIOSOURCE, San Diego, USA). Three human serums were used for negative selection and CRP expressions of the serum samples were under the detection limit (<0.02 μM, Beckman DxC analyzer, Beckman Corporation, Fullerton, CA). Magnetic nanoparticles were bio-functionalized by coating streptavidin on the outermost surface and were dispersed in PBS (pH = 7.4) to form a SA-MNP reagent (purchased from Magqu, Taipei, Taiwan). The average hydrodynamic diameter of the streptavidin-coated magnetic nanoparticles (SA-MNPs) in the reagent was 50 nm. The reagent had a concentration of SA-MNPs with 0.3 emu/g. The biotinylation kit (EZ-Link Sulfo-NHS-Biotinylation Kit) was purchased from Pierce (Rockford, IL, USA). A 200 μl of serum for each negative sample serum was individually incubated with over dosed latex particles, which consisted of a polystyrene core and a hydrophilic shell. These were covalently bonded with anti-CRP monoclonal antibodies (Siemens Health- care Diagnostics, Eschborn, Germany). CRP in the serums formed antigen-antibody complexes with the latex particles. After centrifugation (10,000 rpm for 5 minutes), the CRP in the serum was removed and the supernatant was collected and identified as negative serums. 200 μg of pure CRP protein (for positive selection) and negative serum proteins (for negative selection) were biotinylated separately, according to manufacturer instructions. Biotinylated samples (positive and negative samples) were incubated with a 50 μl of SA-MNP reagent. The high-affinity binding between the streptavidin and biotin ensured conjugation between magnetic nanoparticles, biotinylated CRP, and serum proteins to form CRP-MNP (P1) and negative serum-MNP (N1: negative serum-MNP-1, N2: negative serum-MNP-2 and N3: negative serum-MNP-3) reagents, respectively. As needed, the CRP-MNPs or negative serum-MNPs were obtained from a CRP-MNP reagent and negative serum-MNP reagents, respectively, through magnetic separation. The collected CRP-MNPs or negative serum-MNPs were washed three times with a binding buffer (BD buffer: 50 mM NaH_2_PO_4_, pH 8.0; 150 mM NaCl; 5 mM KCl; 2 mM MgCl_2_; and 0.05% (v/v) Tween-20) and collected with a magnetic stand. The preparation of CRP-coated bio-functionalized magnetic nanoparticles (CRP-MNPs) has been described in Lai *et al*^23^,^24^. All clinical serum samples were stored at −20 °C for further analysis. Clinical serums were obtained from Chung Shan Medical University Hospital (Taichung, Taiwan) with IRB approval (CS15071). All experiments, procedures, and methods were carried out in accordance with the IRB approved guidelines and regulations.

### Experimental Setup

This experimental setup is identical to that of ref. 23 where the detail working principle has been described. The RO-MARAS method uses a rotating magnetic field generated by two sets of Helmholtz coils placed orthogonally. Two signals, cos (*ωt*) and sin (*ωt*), are fed into a 2-channel power amplifier through a LABVIEW program with a NI BNC-2110 capture box. The two signals are amplified equally, driving the coil sets to produce rotating magnetic fields. The sample was placed in the intersection between the central lines of the two sets of Helmholtz coils and the field strength was calibrated using a gauss meter.

### Primer-free MARAS selection process

**Figure 5:**
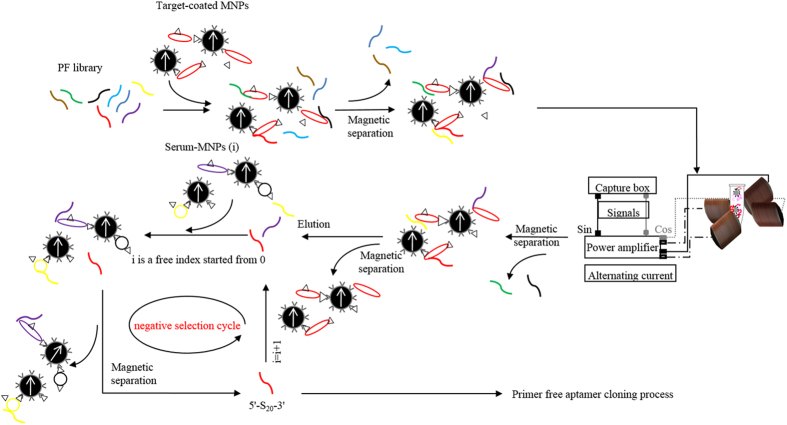
Schematic illustration of the PF-MARAS procedure. The target and negative serum-MNPs (e.g., 1, 2, and 3) were prepared. After the formation of secondary structures, the 20-nt primer free library (5′-N_20_-3′: PF library) was incubated with the target-MNPs. The unbound PF library was removed through magnetic separation. The bound mixture was re-dispersed and subjected to a MARAS selection process. Another magnetic separation was performed to remove the detached oligonucleotides. The bound mixture was re-dispersed and the oligonucleotides were eluted from MNPs through a heating process and then purified. The obtained oligonucleotides were incubated with negative serum-MNPs (1) then a magnetic separation was performed to remove the bound mixture containing oligonucleotides bound to negative MNPs (1) and the supernatant was collected. The collected supernatant containing unbound oligonucleotides was incubated with the next negative serum-MNPs (2). The negative selection process was repeated. After a final run of the negative selection, the final supernatant containing unbound oligonucleotides (PF-aptamer: 5′-S_20_-3′) were obtained, in which, i is a free index starting from 0.

The schematic of primer free RO-MARAS is depicted in Fig. 5 and the detailed process is described in this section. A 20 nt of non-fixed region randomized oligonucleotide library was used as the starting library (PF library). A 5 μM PF library was dissolved in BD buffer and diluted to 10 μl using a BD buffer in a micro-tube. This was heated to 95 °C for 5 minutes and then cooled quickly at 4 °C to form secondary structures, and then brought to room temperature for 30 minutes. A positive selection was performed by adding CRP-MNPs (P1), using magnetic separation from 5 μl of CRP-MNP reagent, into the micro-tube and incubated with the PF library for 30 minutes at room temperature. The unbound nucleotides were removed with a magnetic stand and the bound mixture was washed twice with 1 ml of BD buffer. A 100 μl of BD buffer was added to re-disperse the bound mixture in the micro-tube, which was placed in the RO-MARAS platform. The bound mixture was then subjected to a rotating magnetic field, with a frequency of 27 KHz and strength of 14 gauss, for 10 minutes at room temperature. In order to avoid agglomeration due to the action of the magnetic field on the magnetic nanoparticles, the bound mixture was stirred by pipetting every 2.5 minutes. The purpose of applying a rotating magnetic field to the sample was to generate a competitive mechanism for bindings among the oligonucleotide sequences and the CRP-MNPs. The sequences bound to the CRP-MNPs did not dissociate until the competitive mechanism counteracted the binding strength of sequences toward the CRP-MNPs. Note that an alternating magnetic field can also be used for a positive selection with a setup depicted in ref. 24. Magnetic separation was performed to remove supernatant and the bound mixture was retained. The retained bound mixture was washed twice with BD buffer and re-suspended in 100 μl BD buffer. The bound oligonucleotides in the mixture were eluted from the CRP-MNPs by heating to 95 °C for 5 minutes. Magnetic separation was performed to collect the supernatant and brought to room temperature for 30 minutes. As collected supernatant contained eluted oligonucleotides and CRP, which were detached from magnetic particles during the heating process, a DNA Extraction Miniprep System (Viogene, Taipei, Taiwan) was used to remove CRP from the supernatant and eluted oligonucleotides were re-dispersed in 20 μl of BD buffer. The re-dispersed solution was heated to 95 °C for 5 minutes and cooled quickly at 4 °C. Subsequently, a negative selection was performed by incubating the solution containing eluted oligonucleotides in the supernatant with the negative serum-MNP-1 (N1), using magnetic separation from 5 μl of negative serum-MNP-1 reagent, for 30 minutes at room temperature. After magnetic separation, oligonucleotides bound with negative serum-MNP-1 were removed. The collected supernatant was then incubated with negative serum-MNP-2 (N2) and negative serum-MNP-3 (N3) and magnetically separated to remove the bound mixture sequentially. Then, the obtained primer-free aptamer (5′-S_20_-3′) in the final collected supernatant, which cannot bind to all proteins in negative serums (N1, N2 and N3) except CRP, were purified with a DNA Extraction Miniprep System and dispersed in 16 μl of ddH_2_O. Note, the purpose of multiple rounds of negative selection in the procedure was designed to minimize the possibility of non-specific bindings during immunoassay by removing target-bound aptamers that can bind with other biomolecules in serums during clinical diagnoses. By nature, the more the negative selection round is performed; the higher the specificity is toward the target of selected aptamers during immunoassay. Moreover, even though the concentration of target protein in negative serums might be under the detection limit, it is suggested that the target content in the negative serums is removed prior to negative selection. The level of target removal will affect the efficiency of this protocol due to the elimination of target-bound aptamers by target existed in the negative serums.

### Primer-free aptamer cloning process

**Figure 6:**
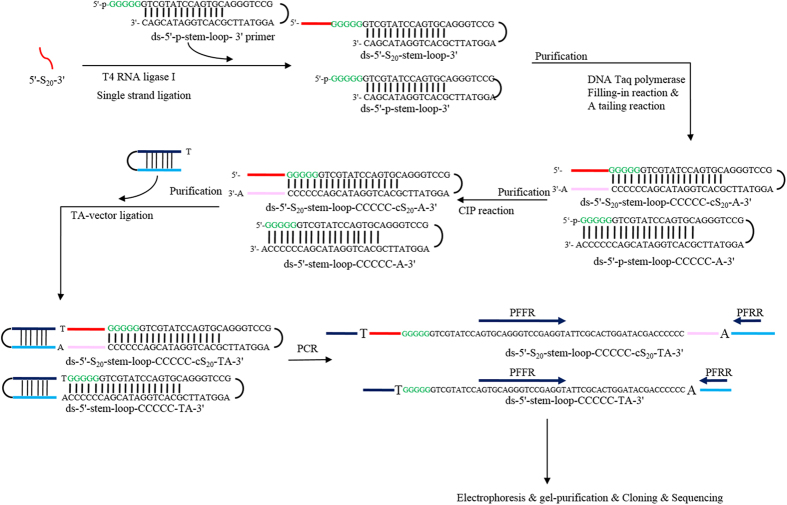
Schematic illustration of the cloning process for primer free aptamers. The 5′-S_20_-3′ was ligated to ds-5′-p-stem-loop-3′ primer by T4 RNA ligation. After purification, an extension was performed by incubated DNA polymerase and dNTP with ligated product. A CIP reaction was performed to remove the 5′-phosphorylation of un-ligated ds-5′-p-stem-loop-3′ primer. After another purification, the ds-5′-S_20_-stem-loop-CCCCC-cS_20_-A-3′ and ds-5′-stem-loop-CCCCC-A-3′ were ligated to TA-vector, and subjected to PCR amplification. A gel electrophoresis was performed for the PCR product, and the gel with correct size of PCR product was cut and used for cloning and sequencing to identify the sequence of PF-aptamer.

As the concentration of obtained aptamers was too low to perform the cloning process, a PCR amplification was necessary to increase the concentration and to generate double strand oligonucleotide sequences. Both forward and reverse primers were needed to perform PCR amplification. However, the sequences of obtained PF- aptamers were not known, which caused difficulty in the following PCR amplification, cloning, and sequencing processes. Therefore, a step to add a known sequence of adapters to aptamers for PCR amplification was necessary. For this purpose, as shown in Fig. 6, single-strand ligation was performed. The reaction was performed in a 20 μl of final volume containing: 5′-S_20_-3′ in 16 μl ddH_2_O, 0.5 mM ds-5′-p-stem-loop-3′ primer(5′-p-GGGGGGTCGTATCCAGTGCAGGGTCCGAGGTATTCGCACTGGATACGAC-3′), 10 U T4 RNA ligase I (New England Biolabs, Beverly, MA, USA), and reaction buffer (50 mM Tris-HCl, 10 mM MgCl_2_, 1 mM DTT, 20% (w/v) polyethylene glycol (PEG)-8000, 1 mM ATP and 2 mM MnCl_2_). Reactants were incubated for 4 hours at 37 °C and deactivated for 15 minutes at 65 °C. A purification process was performed with a DNA Extraction Miniprep System to remove the T4 RNA ligase I and reaction buffer, and to recover ligated DNA aptamers (ds-5′-S_20_-stem-loop-3′) and remaining non-ligated stem-loop primers (ds-5′-p-stem-loop-3′ primer). Both ligated DNA aptamers and non-ligated stem-loop primers were dispersed in 16 μl of ddH_2_O. Consequently, a fill-in reaction was performed in 20 μl of final volume containing: ds-5′-S_20_-stem-loop-3′, ds-5′-p-stem-loop-3′ primer, and 5X PCR Mastermix (Genemark, Taichung, Taiwan) at 72 °C for 30 minutes. A purification process was performed with a DNA Extraction Miniprep System to remove enzyme and reaction buffers, and to recover adenine tailing double strand ligated DNA aptamers, ds-5′-S_20_-stem-loop-CCCCC-cS_20_-A-3′. The 5 Cs and cS_20_ complement the 5 Gs at the 5′ end of the stem-loop primer and S_20_, respectively. Note that the fill-in reaction also caused the formation of ds-5′-p-stem-loop-CCCCC-A-3′ from non-ligated stem-loop primers. The purified nucleic acids including ds-5′-S_20_-stem-loop-CCCCC-cS_20_-A-3′ and ds-5′-p-stem-loop-CCCCC-A-3′ were dispersed in 17 μl of ddH_2_O. In order to prevent ligation of non-ligated stem loop primers (ds-5′-p-stem-loop-CCCCC-A-3′) to other oligonucleotides, a calf intestinal alkaline phosphatase (CIP) reaction was performed to remove 5′ phosphate of ds-5′-p-stem-loop-CCCCC-A-3′ primer. The CIP reaction was performed in a 20 μl of final volume containing: ds-5′-S_20_-stem-loop-CCCCC-cS_20_-A-3′, ds-5′-p-stem-loop-CCCCC-A-3′ primer, 10 U intestinal alkaline phosphatase (New England Biolabs, Beverly, MA, USA), and Cutsmart buffer (50 mM Potassium Acetate, 20 mM Tris-acetate, 10 mM Magnesium Acetate, 100 μM BSA, pH 7.9). Reactants were incubated for 1 hour at 37 °C and then deactivated for 15 minutes at 65 °C. A DNA purification process was performed to remove enzyme and reaction buffer, and to recover ds-5′-S_20_-stem-loop-CCCCC-cS_20_-A-3′ and ds-5′-stem-loop-CCCCC-A-3′. The recovered ds-5′-S_20_-stem-loop-CCCCC-cS_20_-A-3′ and ds-5′-stem-loop-CCCCC-A-3′ were dispersed in 15 μl of ddH_2_O and sub-cloned into a yT&A-vector (Yeastern Biotech, Taipei, Taiwan) through DNA ligation, which was performed in accordance to manufacturer instructions. A PCR amplification was performed using a set of primers: PFFR and PFRR for amplifying partial sequences containing cS_20_ of the obtained ds-5′-S_20_-stem-loop-CCCCC-cS_20_-TA-3′. Also, a partial sequence of ds-5′-stem-loop-CCCCC-TA-3′ ligated with yT&A-vector was also amplified. The PCR reaction, which contained 1.25 U of DNA polymerase (Invitrogen), 0.1 mM of dNTPs, 0.5 mM of MgSO_4_, and 0.5 nM of primers, was performed under the following conditions: 30 cycles at 40 seconds at 94 °C; 40 seconds at 60 °C, and 40 seconds at 72 °C. The PCR amplification increased the number of specific sequences in ligated ds-5′-S_20_-stem-loop-CCCCC-cS_20_-TA-3′, as well as in non-ligated ds-5′-stem-loop-CCCCC-TA-3′. The PCR product was mixed with 6X DNA loading dye (0.25% bromophenol blue, 0.25% xylene cyanol, and 15% Ficoll) and the sample was run using a 3% agarose gel. The electrophoresis result was analyzed by Alpha Imager EC (Alpha Innotech Corporation, San Leandro, CA, USA) and the full-length gel is presented in Supplementary Fig. S4. There are two PCR products: a 64 bp (35 + 5 + 24) in which no aptamer ligated to stem loop primer and the other is 84 bp (35 + 5 + 20 + 24), which is the corrected one with ligated aptamer. The length of the partial stem-loop primer counting from PFFR primer at 5′ end was 35, the length of 5 Cs complemental to the first 5 Gs at 5′ end of stem-loop primer was 5, the length of cS_20_ was 20, and the length of monomers within yT&A-vector counting from PFRR was 24. Note that if other partial sequences of stem-loop primer and other partial sequences within the yT&A-vector are used to replace PFFR and PFRR, respectively, the sizes of PCR product will change accordingly. The PCR product of the corrected size was cut from the agarose gel, recovered with a DNA Extraction Miniprep System and dispersed in 15 μl of ddH_2_O. The recovered PCR product was sub-cloned into yT&A-vector, and transformed into DH5α competent cells. The set of primers, M13FR and M13RR, was used to verify the cloning step. Randomly-chosen plasmid clones from the experiment were further analyzed. The plasmids of the randomly selected clones were purified using a High-Speed Plasmid Mini Kit (Geneaid, Taipei, Taiwan). The plasmids were sequenced using an Applied Biosystems PRISM 3730 DNA automatic sequencer and a Big Dye terminator cycle sequencing kit (Applied Biosystems, Foster City, CA, USA). The secondary structures of the aptamers were predicted using an Mfold program^25^.

### Reverse validation of selected aptamer

**Figure 7:**
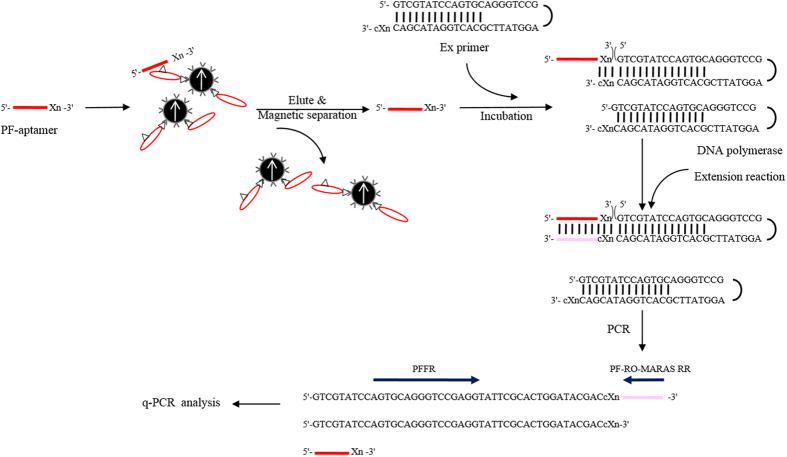
Schematic diagram illustrating the experimental procedure of reverse transcription for determining binding specificity of selected PF-aptamers toward target.

To validate the binding specificity of the selected PF-aptamer, 100 nM of the selected aptamer was heated in 20 μl of BD buffer to 95 °C for five minutes and cooled at 4 °C to form secondary structures. The aptamers were individually incubated with CRP-MNPs (P1; positive control) and negative serum-MNPs (N1, N2 and N3; negative controls), obtained from 5 μl of corresponding reagents through magnetic separation, respectively, for 30 minutes at room temperature. The supernatant was removed through magnetic separation and the bound mixture was collected. The bound mixture was re-dispersed with 200 μl of BD buffer. Half (100 μl) of the bound mixture solution was subjected to magnetic separation and the retained bound mixture was re-dispersed in 100 μl of ddH_2_O. The re-dispersed bound mixture was identified as “Before NSS”. The other half of bound mixture solution was placed inside RO-MARAS platform, and then subjected to a rotating magnetic field, with a frequency of 27 KHz and strength of 14 gauss, for 10 minutes at room temperature. A magnetic separation was performed to remove supernatant and the bound mixture was re-dispersed in 100 μl of ddH_2_O, which was after the application of nonspecific suppression magnetic field and then identified as “After NSS”. The “Before NSS” and “After NSS” solutions were heated to 95 °C for 5 minutes to elute aptamers from the CRP-MNPs and negative serum-MNPs. Magnetic separation was performed to remove the MNPs and collect supernatant. The eluted aptamers in the supernatant were precipitated with ethanol. The precipitated aptamers were re-dispersed in 100 μl of ddH_2_O. Analysis of the amount of the aptamers binding with the target was measured by a real-time quantitative PCR (q-PCR). However, the isolated aptamer without primer-recognized region could not be used as a template for q-PCR analysis. Therefore, a reverse transcription in combination with a corresponding sequence of stem-loop primer was performed. The schematic diagram of reverse transcription reaction of the aptamer is illustrated in Fig. 7. For the reverse transcription, an extension reaction was performed for the eluted aptamers. For the extension, the previously described Ex primer and cXn in the Ex primer is complemental to Xn at the 3′ end of the corresponding aptamer, where Xn is n monomers of 5′-S_20_-3′ counting from the 3′ end. Note that the length (n) of nucleotide monomers at the 3′ end of the Ex primer were maintained to ensure the correct extension reaction. The extension reaction was performed with the re-dispersed aptamer (5′-S_20_-3′) of 15 μl of 5X PCR Mastermix and 10 μM of the corresponding Ex primer (totaling 20 μl). The reaction condition follows: incubation at 54 °C for 10 minutes in which the Ex primer was annealed to the isolated aptamer according to complementary sequence regions and then at 72 °C for 30 minutes to extend nucleic acid. The final solution contained three spices: the obtained aptamer (5′-S_20_-3′), Ex primer, and the extended Ex primer with cS_20_ at the 3′ end. The final product was diluted (5X) and the quantities of the nucleic acids (corresponding to eluted aptamers) were calculated with a SYBR based q-PCR reaction. The mixture for each q-PCR run was 10 μl, containing 5 μl of diluted nucleic acids, 2.5 μl of SYBR Green PCR master mix (Applied Biosystems), and 0.5 nM of primers. The reaction condition was as follows: 95 °C for 3 minutes; 40 cycles at 94 °C for 30 seconds; 60 °C for 30 seconds; and 72 °C for 30 seconds. The primers, PFFR and PF-RO-MARAS RR, were used for q-PCR to amplify the diluted nucleic acids. PFFR primer is common to the q-PCR analysis for all the aptamers. PF-RO-MARAS RR primer contained sequences of isolated aptamer, which excluded monomers of Xn at the 3′ end. With the set of primers used, only the extended Ex primer with cS_20_ at its 3′ end was amplified during q-PCR reaction.

### Determination of equilibrium dissociation constants by q-PCR

The equilibrium dissociation constant (K_d_) of selected aptamers for the target was measured by a real-time quantitative PCR (q-PCR)^23^,^24^. The process is as follows: a series of progressively diluted aptamers (200 nM to 1.5625 nM) in 20 μl of BD buffer were heated to 95 °C for five minutes and cooled at 4 °C to form secondary structures. A partial series of diluted aptamers were retained for input control (Input). CRP-MNPs, obtained through magnetic separation from 5 μl of CRP-MNP reagent, was added into each diluted aptamer tube and incubated for 30 minutes at room temperature. The supernatant was removed with a magnetic stand and the bound mixture was collected. The bound aptamers were eluted from the MNPs by applying heat at 94 °C for 10 minutes in a final volume of 20 μl of ddH_2_O. The MNPs were removed through magnetic separation. Both the input controls and eluted aptamers were precipitated with ethanol and dissolved in 100 μl of ddH_2_O. A reverse transcription was performed for dissolved aptamers, as described above in Fig. 7, and then a q-PCR analysis was conducted, as described above. The apparent dissociation constants (K_d_) of selected aptamers were determined by quantitating the nucleic acid resulting from the reverse transcription by q-PCR and fitting the results in a non-linear regression. The concentrations of nucleic acids in each input control and eluted nucleic acids were calculated using a 200 nM concentration of nucleic acids, as indicative of maximum binding. The K_d_ value of the selected aptamer (5′-S_20_-3′) was determined by fitting a saturation binding curve based on experimental data through a curve fitting program, CurveExpert1.3 (curveexpert.webhop.net). The K_d_ value of the selected aptamer was performed in duplicate for each q-PCR run and expressed as the mean ± standard deviation from the three separate experiments performed.

### Establishing the standard curve by q-PCR

Standard calibration curves were determined by using a serial dilution of CRP-MNPs, obtained from 1 μl of CRP-MNP reagent through magnetic separation in 1 μl of BD buffer. The corresponding CRP concentrations in the diluted solution were 4000, 2000, 1000, and up to 31.25 nM. For each CRP concentration, 1 μM of PF-aptamer (PF20N-RO-MARAS-84-1) in 10 μl of BD buffer solution which was heated to 95 °C for five minutes and cooled at 4 °C to form secondary structures, was incubated with CRP-MNPs, obtained through magnetic separation from the diluted solution, for 30 minutes at room temperature. The bound mixture was collected and the supernatant was removed with a magnetic stand. The bound aptamers were eluted from the MNPs by heat at 94 °C for 10 minutes in a final volume of 100 μl of ddH_2_O and the MNPs were removed through magnetic separation. q-PCR analyses were performed in duplicate, as described above, for each collected supernatant. The CRP concentration, represented by the relative expression level resulted from q-PCR analysis, was determined from the PCR cycle number at which fluorescence intensity reaches a set cycle threshold value (ct). The standard curve was linearly fitted from sixteen measured data points to obtain the linear equation and R^2^ value. The standard calibration curve was used to determine sample concentrations for future analyses.

### Recovery rate of spiked CRP using PF-aptamer as a capture probe

Additional three clinical serums were used to perform recovery rate analysis, of which CRP concentrations were low (<0.02 μM measured by Nephelometry analysis). The corresponding serum-MNPs without CRP were prepared, as discussed above (identified as serum-1, -2, and -3 MNPs). For the recovery rate analysis, 5000 nM of the selected aptamer was dispersed in 20 μl of BD buffer and was heated to 95 °C for five minutes and cooled at 4 °C to form secondary structures. CRP-MNPs (4000 ng, CRP protein), obtained from magnetic separation of 1 μl of CRP-MNP reagent, were mixed with serum-1, -2, and -3 MNPs separately to form correspondingly mixed MNPs. Then the CRP-MNPs (severed for a control) and mixed MNPs were added into the BD buffer containing selected aptamers and incubated for 30 minutes at room temperature. The supernatant was removed with a magnetic stand and the bound mixture was collected and dispersed in 200 μl of BD buffer. Half of the bound mixture solution (100 μl) was subjected to magnetic separation to remove supernatant and the bound mixture was collected and dispersed in 100 μl of ddH_2_O, identified as “Before NSS”. The other half of bound mixture solution (100 μl) was placed inside a RO-MARAS platform, as discussed previously. Magnetic separation was performed to remove supernatant and the bound mixture was re-dispersed in 100 μl of ddH_2_O, identified as “After NSS”. Both “Before NSS” and “After NSS” solutions were heated to 95 °C for 5 minutes to elute aptamers from the MNPs. Magnetic separation was performed to remove MNPs and collect supernatant. CRP concentrations in control and serum samples were analyzed by q-PCR with a linear equation determined by the standard calibration curve.

### PF-aptamer as a bio-probe in assaying blind serum samples

Forty clinical serums were collected through the preparation of blind sample bio-functional magnetic particles (blind-MNPs) without the removal of CRP, as described above. 2000 nM of selected aptamer in 20 μl of BD buffer was heated to 95 °C for five minutes and cooled at 4 °C to form secondary structures. Aptamers were individually incubated with blind-MNPs, obtained from 2 μl of blind-MNP reagent through magnetic separation, for 30 minutes at room temperature. The supernatant was removed with a magnetic stand and the bound mixture was collected and re-dispersed in 200 μl of BD buffer. Half of the bound mixture solution (100 μl) was subjected to magnetic separation to remove supernatant and the bound mixture was collected and dispersed in 100 μl of ddH_2_O, identified as “Before NSS”. The other half of the bound mixture solution (100 μl) was placed inside a RO-MARAS platform, as discussed previously. Magnetic separation was performed to remove supernatant and the bound mixture was re-dispersed in 100 μl of ddH_2_O, identified as “After NSS”. Both “Before NSS” and “After NSS” solutions were heated to 95 °C for 5 minutes to elute aptamers from the blind-MNPs. Magnetic separation was performed to remove MNPs and collect supernatant. CRP concentrations of blind samples were determined by q-PCR through the linear equation of the standard calibration curve. Additionally, concentrations of CRP in blind serum samples were measured by monoclonal antibody based Nephelometry analysis (Siemens Health- care Diagnostics, Eschborn, Germany). The results of the blind serum samples determined by q-PCR, using PF-aptamer as a bio-probe, were compared to those using monoclonal antibody-based Nephelometry. To determine the correlation of measured results between two assaying methods, Spearman correlation coefficients and associated *P* values were calculated. Spearman’s rho analysis was applied with SPSS software (Version 13.0 SPSS Inc., Chicago, IL), for which P < 0.05 was considered statistically significant. The Bland-Altman plot was used to compare two methods with EXCEL software (Office 2013). We calculated the mean difference between the monoclonal antibody-based nephelometry and MARAS methods, including “Before NSS” and “After NSS”. The R-R interval measurements (bias) and the 95% limits of measurement (bias ± 1.96 SE) were analyzed for comparing the two methods.

## Additional Information

**How to cite this article:** Tsao, S.-M. *et al*. Generation of Aptamers from A Primer-Free Randomized ssDNA Library Using Magnetic-Assisted Rapid Aptamer Selection. *Sci. Rep.* **7**, 45478; doi: 10.1038/srep45478 (2017).

**Publisher's note:** Springer Nature remains neutral with regard to jurisdictional claims in published maps and institutional affiliations.

## Supplementary Material

Supplementary Information

## Figures and Tables

**Table 1:** Sequence of oligonucleotides.

Oligo	5′-Sequence-3′
PF20N-RO-MARAS-84-1	GTTGACGGGCGATTGGTCTT
PF20N-RO-MARAS-84-1 Ex primer	GTCGT ATCCA GTGCA GGGTC CGAGG TATTC GCACT GGATA CGACAAGACC
PF20N-RO-MARAS-84-1 RR primer	GTTGACGGGCGATT

**Table 2:** Comparison between primer-free RO-MARAS (PF-RO-MARAS) and modified primer-free SELEX (PF-SELEX).

Characteristics	PF-RO-MARAS	Other modified PF-SELEX*
Require time (weeks)	1<	4~12
Evolution cycle (rounds)	1	4~15
Waste Cost	Low	High
Technical difficulty	Moderate	High
Aptamer produce yield	Low	Low

*Other modifications of primer-free SELEX were referred from references 16, 17, 18, 19, 20, 21, 22.
