# Supplementary material for: Generation of Aptamers from A Primer-Free Randomized ssDNA Library Using Magnetic-Assisted Rapid Aptamer Selection
Source: Sci Rep. 2017 Apr 3;7:45478. doi: 10.1038/srep45478 (PMC5377317; doi:10.1038/srep45478)
Supplement: Supplementary Information [file srep45478-s1.pdf]

**Generation of Aptamers from A Primer-Free Randomized ssDNA Library Using Magnetic-Assisted Rapid Aptamer Selection**

Shih-Ming Tsao, Ji-Ching Lai, Horng-Er Horng, Tu-Chen Liu, Chin-Yih Hong

Table of contents

Table S1. Sequence of aptamers.

Fig. S1. The secondary structure of selected PF-aptamers (PF20N-RO-MARAS-84-1) predicted by MFold analysis.

Fig. S2. The secondary structure of PF20N-RO-MARAS-84-1 Ex primer predicted by MFold analysis.

Fig. S3. Representative duplicated results of dissociation constant of selected PF-aptamer (a). Kd: 24.16 nM, (b). Kd: 23.00 nM.

Fig. S4. Agarose gel electrophoresis of PCR product.

Table S1. Sequence of aptamers

| Oligo | 5'-Sequence-3' |
| --- | --- |
| PF20N-RO-MARAS-84-2 | CACAGCTAGAATAAGTGCGT |
| PF20N-RO-MARAS-84-3 | AACAAAACAATATCAGCTAT |
| PF20N-RO-MARAS-84-6 | TCTGTCCCTGGCTATAACCT |
| PF20N-RO-MARAS-84-7 | CAAAGATACTAGTCATGTCA |

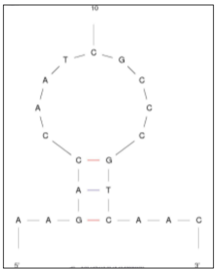

ΔG = -2.93 kcal/mol

Fig. S1. The secondary structure of selected PF-aptamers (PF20N-RO-MARAS-84-1) predicted by MFold analysis.

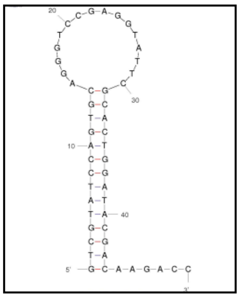

Fig. S2 The secondary structure of PF20N-RO-MARAS-84-1 Ex primer predicted by MFold analysis.

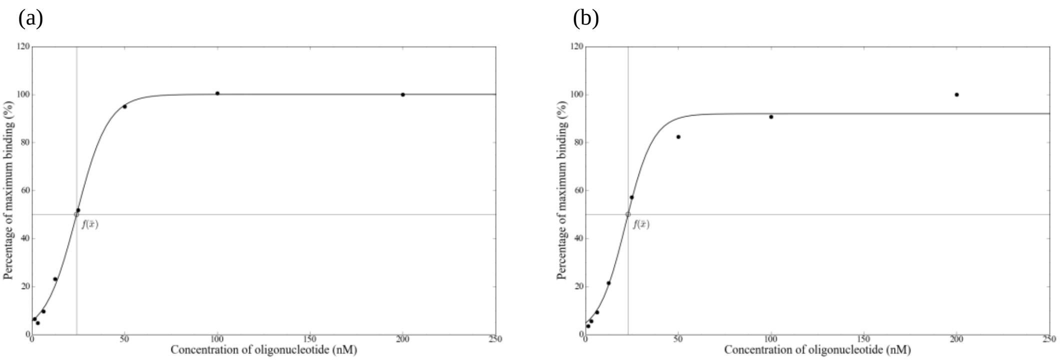


Fig. S3. Representative duplicated results of dissociation constant of selected PF-aptamer (a). $K_d$: 24.16 nM, (b). $K_d$: 23.00 nM.

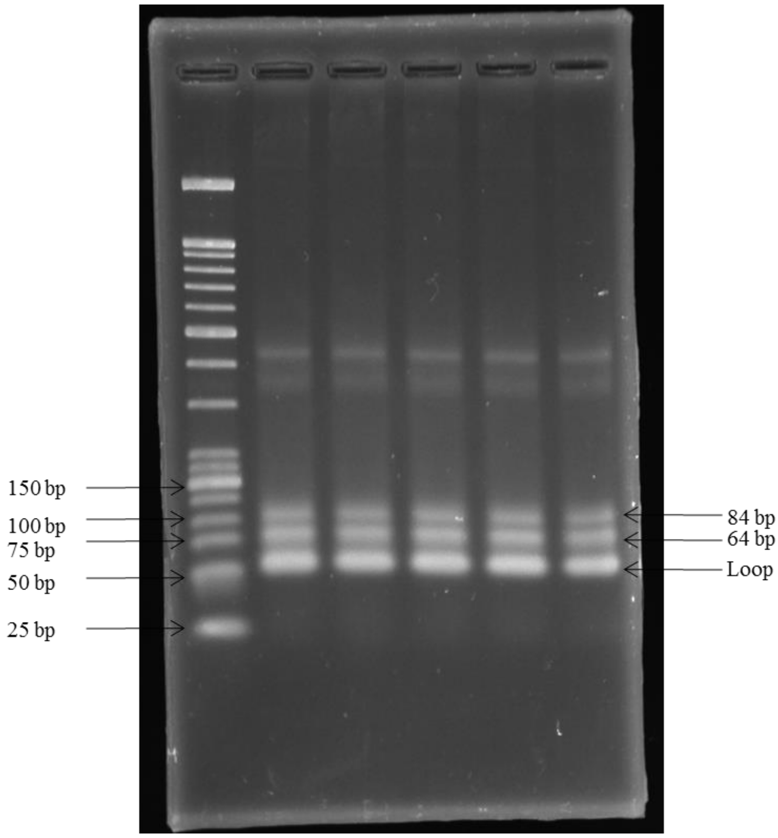


Fig. S4. Agarose gel electrophoresis of PCR product.
